# MiR-409-3p Inhibits Cell Proliferation and Invasion of Osteosarcoma by Targeting Zinc-Finger E-Box-Binding Homeobox-1

**DOI:** 10.3389/fphar.2019.00137

**Published:** 2019-02-21

**Authors:** Liang Wu, Yiming Zhang, Zhongyue Huang, Huijie Gu, Kaifeng Zhou, Xiaofan Yin, Jun Xu

**Affiliations:** Minhang Hosptial, Fudan University, Shanghai, China

**Keywords:** osteosarcoma, microRNA-409, molecular mechanism, zinc-finger E-box-binding homeobox-1, invasion

## Abstract

Osteosarcoma (OS) is the most common bone cancer worldwide. There is evidence that microRNA-409 (miR-409-3p) is involved in tumorigenesis and cancer progression, however, its possible role in OS requires clarification. In the present study, we evaluated the expression level, clinical significance, and mode of action of miR-409-3p in OS. The miR-409-3p levels were diminished in the OS cells and tissues compared with associated adjacent non-tumor tissues and a non-cancer osteoplastic cell line. Low miR-409-3p expression levels were associated with clinical stage and distant metastasis in patients with OS. Resumption of miR-409-3p expression attenuated OS cell proliferation and invasion. Additionally, based on informatics analyses, we predicted that zinc-finger E-box-binding homeobox-1 (ZEB1) is a possible target of miR-409-3p. This hypothesis was confirmed using luciferase reporter assays, reverse transcription-quantitative real-time polymerase chain reaction, and Western blot analyses. The findings of the current study indicated that ZEB1 was up-regulated in the OS tissues and cell lines, and that this up-regulation was inversely proportional to miR-409-3p expression levels. Furthermore, down-regulation of ZEB1 decreased OS cell invasion and proliferation, illustrating that the tumor suppressive role of miR-409-3p in OS cells may be exerted *via* negative regulation of ZEB1. Taken together, our observations highlight the potential role of miR-409-3p as a tumor suppressor in OS partially through down-regulation of ZEB1 and suggest that miR-409-3p has potential applications in OS treatment.

## Introduction

Osteogenic sarcoma (osteosarcoma; OS) is among the most common forms of bone cancer globally. The incidence ranges from 4 to 5 cases per million among children and teenagers (Gill et al., 2013; Tang et al., 2014). The OS tumors are always located in the distal femur or proximal tibia, and tumors in these regions present a high tendency to destroy adjacent normal tissues (Cates, 2016; Righi et al., 2016). Despite considerable advances in treatment strategies such as surgery, radiotherapy, chemotherapy and new antineoplastic agent (Li et al., 2015), cases with metastatic or recurrent OS have an inferior prognosis, and the likelihood of long-term survival for patients with advanced OS remains very low (Anderson, 2015; Isakoff et al., 2015). Genetic and epigenetic variations and potential environmental factors that block mesenchymal stem cell differentiation into osteoblasts contribute to OS tumorigenesis and tumor development (Sun et al., 2015; Li et al., 2018; Zhang et al., 2018), however, the detailed and complex molecular mechanisms underlying OS development remain largely unknown. Therefore, the molecular mechanisms underlying OS formation and progression require investigation to facilitate the development of novel therapeutic approaches for application in patients with OS.

MicroRNAs (miRNAs) are a subtype of endogenous, non-coding, single-stranded, short RNAs, with an approximate range in length of 19–25 nucleotides (Esteller, 2011). miRNAs can regulate the expression of protein-coding genes by binding to complementary sequences in the 3′-untranslated regions (3′ UTRs) of target genes, causing translational repression or mRNA cleavage (Bartel, 2004). miRNAs play key roles in various cellular processes, including apoptosis, cell proliferation, differentiation, angiogenesis, invasion, and metastasis (Gambari et al., 2016). Recently, the abnormal expression of miRNAs has been implicated in the etiology and development of various human cancers (Chen et al., 2016; Li and Wang, 2016; Wang et al., 2016). The potential biological roles of several miRNAs abnormally expressed in OS during its tumorigenesis have also been highlighted. For example, miR-422a expression is down-regulated in OS cell lines and tissues. Conversely, high levels of miR-422a expression can suppress OS cell invasion and proliferation, and improve paclitaxel and cisplatin-mediated apoptosis (Liu et al., 2016). Therefore, there is a need to explore the potential role of miRNA expression in OS and to unravel the underlying primary molecular mechanisms, which may provide information useful for designing new and efficient therapeutic strategies aimed at curing OS.

The effect of miR-409-3p has been investigated in various human malignancies, including breast (Ma et al., 2016), gastric (Zheng et al., 2012), colon (Tan et al., 2016), and prostate (Josson et al., 2015) cancers, however, its role of miR-409-3p in OS remains unclear. Latest study confirmed the interaction of miR-409-3p and ZEB1 played a role in the progression process of non-small cell lung cancer, indicating ZEB1 acted as a direct target of miR409-3p and could be modulated by miR-409-3p (Qu et al., 2018). Herein, we hypothesis there exists the miR-409-3p/ZEB1 axis in OS and report the first investigation of the expression levels, clinical significance, and biological functions of miR-409-3p in OS, as well as its underlying molecular mechanism.

## Materials and Methods

### Ethics Statement

All study participants voluntarily provided written consent before entering the study. We obtained the approval of The Ethics Committee of the Minhang Hospital, Zhongshan Hospital, Fudan University for Disease Control and Prevention. The methodology used in this study completely conformed to the recommendations of CONSORT 2010.

### Tissue Specimens

Forty-nine pairs of osteosarcoma tumor and adjacent non-tumor tissues were collected from patients with osteosarcoma at Minhang Hospital, Zhongshan Hospital, Fudan University. No participants underwent chemotherapy or radiotherapy before surgery. All tissues samples were directly transferred into liquid nitrogen and were stored at -80°C until RNA extraction.

### Cell Lines

OS cell lines, including HOS (GDC76) and MG63 (GDC074) were obtained directly from the Chinese Academy of Medical Sciences (Beijing, China) Cell Resource Center. A non-cancer osteoblastic cell line (hFOB 1.19 CRL-11372) was obtained from the American Type Culture Collection (ATCC; Manassas, VA, United States). All cells were incubated in Dulbecco’s modified Eagle medium (DMEM; Gibco, Invitrogen Life Technologies, Carlsbad, CA, United States) supplemented with 10% fetal bovine serum (FBS; Gibco, Invitrogen Life Technologies, Carlsbad, CA, United States). All experimental cells were maintained at 37°C in 5% (*V*/*V*) carbon dioxide (CO_2_) and passaged every 2–3 days.

Cells were then seeded in 6-well plates at a density of 50–60% confluence for transfection. After overnight incubation, cells were transfected with miR-409-3p mimics, negative control miRNA mimics (miR-NC), ZEB1 siRNA, or scrambled siRNA (GenePharma, Shanghai, China), using Lipofectamine 2000 transfection reagent (Invitrogen, Carlsbad, CA, United States), according to the manufacturer’s guidelines. Post-transfection (6 h), the culture medium was changed to DMEM containing 10% FBS.

### Reverse Transcription-Quantitative Real-Time Polymerase Chain Reaction (RT–qPCR)

RNA was extracted using Trizol reagent (Invitrogen, Carlsbad, CA, United States), following the manufacturer’s directions. A TaqMan Micro-RNA Reverse Transcription Kit (Applied Biosystems, Foster City, CA, United States) was used to reverse-transcribe miRNA, and qPCR performed using a TaqMan Micro-RNA PCR Kit (Applied Biosystems, Foster City, CA, United States). Reverse transcription of mRNA was performed using the M-MLV Reverse Transcription system (Promega Corporation, Madison, WI, United States). To determine *ZEB1* mRNA expression levels we used the primers: forward 5′-AGGCAATAGGTTTTGAGGGCCAT-3′ and reverse 5′-TGCACCTTCTGTCTCGGTTTCTT-3′ and SYBR Premix Ex Taq (TaKaRa, Dalian, China). Endogenous U6 small nuclear RNA (primers: forward, 5′-CTCGCTTCGGCAGCACA-3′; reverse, 5′-AACGCTTCACGAATTTGCGT-3′) was amplified as an internal control for miR-409-3p, and β-actin (primers: forward, 5′-AGCGAGCATCCCCCAAAGTT-3′; reverse, 5′-GGGCACGAAGGCTCATCATT-3′) was amplified as an internal control for *ZEB1* mRNA. All RT-qPCR experiments were conducted using an ABI7500 Real-time PCR system (Applied Biosystems, Carlsbad, CA, United States). Relative mRNA or miRNA expression levels were quantified using the 2^-ΔΔCt^ method (Livak and Schmittgen, 2001).

### 3-(4,5-Dimethylthiazol-2-yl)-2,5-Diphenyltetrazolium Bromide (MTT) Assay

Post-transfection (24 h), cells were re-seeded into 96-well plates at 3,000 per well. Cells were maintained at 37°C in 5% (*V*/*V*) CO_2_ for 4 days. Then, cell proliferation was tested at the indicated times using the MTT assay (Sigma, St. Louis, MO, United States). In brief, 0.5 mg/mL MTT solution was added to cells, which were then incubated at 37°C for a further 4 h. Subsequently, we added 150.0 μL DMSO (Sigma, St. Louis, MO, United States) into each to dissolve the formazan crystals. Spectrometric absorbance was determined using a microplate reader (Bio-Rad Laboratories Inc., Hercules, CA, United States) at a wavelength of 490 nm.

### Cell Invasion Assay

After 48 h transfection, cells were collected and suspended in FBS-free culture medium. Then, 5 × 10^4^ cells were added into upper chambers of a 24-well Transwell Permeable Support device (8-μm pores, Costar; Corning Incorporated, Corning, NY, United States) coated with Matrigel (BD Biosciences, San Jose, CA, United States), while 500.0 μL culture medium containing 20% FBS was added to the lower chambers and cells incubated at 37°C in 5% CO_2_ for 48 h. We removed cells in the upper chambers using cotton swabs, then invaded cells were fixed with methanol, stained with 0.5% crystal violet, washed, and dried in air. An inverted microscope (Olympus Corporation, Tokyo, Japan) (200 × magnification) was used to calculate the number of invading cells in five randomly selected fields.

### Prediction of miR-409-3p Targets and Luciferase Reporter Assays

Two miRNA targeted-gene databases, TargetScan^1^ and miRanda^2^, were used to predict target genes of miR-409-3p. HEK293T cells (ATCC) were seeded into 24-well plates at 40–50% confluence. After 24 h, cells were transfected with miR-409-3p mimics or miR-NC and pmirGLO-ZEB1-3′UTR-mutant (Mut) (GenePharma) or pmirGLO-ZEB1-3′UTR-wild-type (Wt) using Lipofectamine 2000. Cells were maintained at 37°C in 5% (*V*/*V*) CO_2_ for 48 h and luciferase reporter assays conducted using the Dual-Luciferase Reporter Assay System (Promega Corporation, Madison, WI, United States). Renilla luciferase was used as an internal control.

### Western Blot Analyses

Cells were harvested after transfection for 72 h and lysed with RIPA Lysis Buffer (Beyotime Institute of Biotechnology, Haimen, China). Protein concentrations were determined using a BCA assay kit (Pierce^TM^; Thermo Fisher Scientific, Inc.). Equal amounts of protein were separated by SDS-PAGE, transferred to polyvinylidene difluoride membranes (Millipore, MA, United States), blocked with 5% skimmed milk for 2 h at room temperature, then incubated overnight at 4°C with mouse anti-human GAPDH monoclonal antibody (sc-137179; 1:1000 dilution; Santa Cruz Biotechnology) or mouse anti-human ZEB1 monoclonal antibody (sc-81428; 1:1000 dilution; Santa Cruz Biotechnology, CA, United States). Membranes were then washed three times using Tris-buffered saline containing 0.1% Tween-20 and probed with horseradish peroxidase-conjugated secondary immunoglobulin G goat anti-mouse (catalog no, sc-2005; 1:10,000) for 2 h at room temperature. Protein bands were visualized using enhanced chemiluminescence reagents (Bio-Rad Laboratories Inc., Hercules, CA, United States) and band densities analyzed using AlphaEase FC software (version 4.0.1; ProteinSimple, San Jose, CA, United States).

### Statistical Analyses

Data were presented as means ± S.D. or box plots. We used SPSS 17.0 software (SPSS Inc., Chicago, IL, United States) for data analyses. Differences among groups were evaluated using one-way ANOVA corrected for multiple comparisons or Student’s *t*-tests. The χ^2^-test was used to evaluate associations between miR-409-3p expression levels and clinicopathological factors. Spearman’s correlation analysis was used to determine the correlation between miR-409-3p and *ZEB1* mRNA expression levels. All statistical tests were two-sided; *P* < 0.05 were considered statistically significant.

## Results

### MiR-409-3p Was Downregulated in OS Tissues and Cell Lines

**FIGURE 1:**
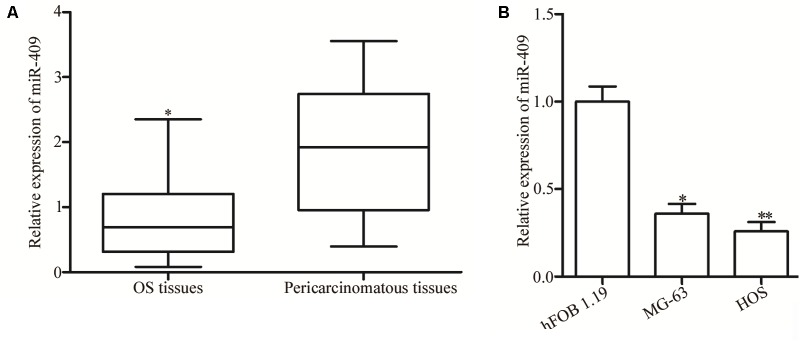
Expression of miR-409-3p in OS tissues and cell lines. **(A)** Relative expression levels of miR-409-3p in 49 paired OS tumor and adjacent non-tumor tissues were evaluated by RT-qPCR. **(B)** Expression of miR-409-3p in OS cell lines compared with that in a non-cancer osteoblastic cell line (hFOB1.19). miR-409-3p, microRNA-409. OS, osteosarcoma. ^∗^*P* < 0.05, ^∗∗^*P* < 0.01 compared with the control group.

RT-qPCR was used to evaluate miR-409-3p expression levels in OS tumor and adjacent non-tumor tissues. Expression of miR-409-3p was lower in OS tissues than that in adjacent non-tumor and normal tissue controls (Figure 1A, *P* < 0.05). Moreover, remarkable low levels of MiR-409-3p expression were detected in two OS cell lines relative to those in a non-cancer osteoblastic cell line (hFOB 1.19) (Figure 1B, *P* < 0.05).

### Relationship Between miR-409-3p Expression and OS Clinicopathological Factors

We also determined the relationship between miR-409-3p expression levels and OS clinicopathological factors. Our data showed that low miR-409-3p expression levels were significantly associated with advanced clinical stage (*P* = 0.035) and distant metastasis (*P* = 0.030), however, there were no significance associations with other clinicopathological factors, including sex (*P* = 0.961), age (*P* = 0.804), and tumor size (*P* = 0.851) (Table 1).

**Table 1:** Correlation of microRNA-409 expression with clinicopathological feature of osteosarcoma.

Variables	Case number	microRNA-409 expression		*P*
		Low	High	
**Sex**				0.961
Male	31	17	14	
Female	18	10	8	
**Age (years)**				0.804
< 20^∗^	21	12	9	
≥ 20	28	15	13	
**Tumor size (cm)**				0.851
< 8	26	14	12	
≥ 8	23	13	10	
**Clinical stage**				0.035^∗^
I-II	23	9	14	
III-IV	26	18	8	
**Distant metastasis**				0.030^∗^
Present	24	17	7	
Absent	25	10	15	


^∗^These participants were above 16 years age. ^∗^*P* < 0.05.

### MiR-409-3p Reduces OS Cell Proliferation and Invasion

**FIGURE 2:**
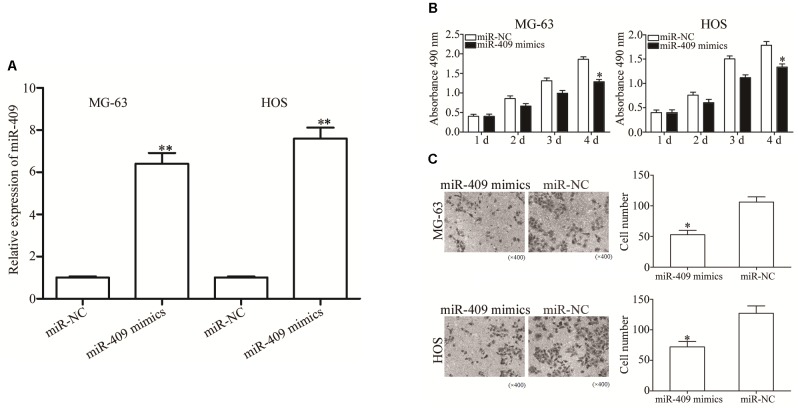
The effects of miR-409-3p overexpression on cell proliferation and invasion in OS. **(A)** Relative expression of miR-409-3p in MG63 and HOS cells following transfection with miR-409-3p mimics or miR-NC. **(B)** MTT assays were performed to assess the effect of miR-409-3p overexpression on MG63 and HOS cells proliferation. **(C)** Cell invasion assays were conducted in MG63 and HOS cells following transfection with miR-409-3p mimics or miR-NC. miR-409, microRNA-409-3p (magnification, ×200). OS, osteosarcoma. miR-NC, negative control microRNA mimics. ^∗^*P* < 0.05, ^∗∗^*P* < 0.01 compared with the control group.

To investigate the role of miR-409-3p in OS, we transfected MG63 and HOS cells with miR-409-3p mimics, and used RT-qPCR to determine miR-409-3p expression levels (Figure 2A, *P* < 0.05). We investigated the role of miR-409-3p in OS cell proliferation using MTT assays conducted in MG63 and HOS cells transfected with miR-409-3p mimics or miR-NC. Expression of miR-409-3p led to a significant decline in MG63 and HOS cell proliferation (Figure 2B, *P* < 0.05). Similarly, the invasion capacity of HOS and MG63 cells transfected with miR-NC or miR-409-3p mimics was estimated using a cell invasion assay. As illustrated in Figure 2C, the introduction of miR-409-3p mimics into HOS and MG63 cells resulted in a considerable decline of invasion ability relative to the miR-NC group (*P* < 0.05). These observations suggested that miR-409-3p has a crucial role in the suppression of OS growth and metastasis.

### A Potential miR-409-3p Target in OS

**FIGURE 3:**
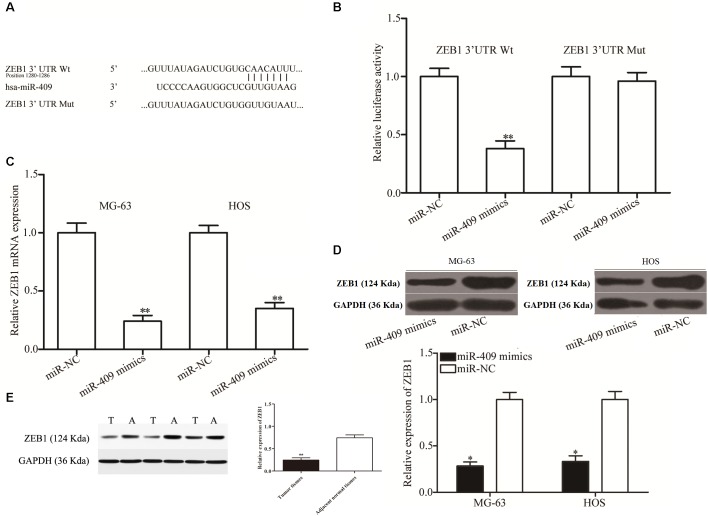
ZEB1 is a direct target of miR-409-3p in OS. **(A)** *ZEB1* 3′ UTR sequences containing wild type and mutant miR-409-3p binding sites. **(B)** Luciferase reporter assays performed in HEK293T cells co-transfected with miR-409-3p mimics or miR-NC, and pmirGLO-ZEB1-3′UTR Wt or pmirGLO-ZEB1-3′UTR Mut. After transfection (48 h), cells were collected and luciferase activities measured. ZEB1 mRNA **(C)** and protein **(D)** were detected in MG63 and HOS cells transfected with miR-409-3p mimics or miR-NC. miR-409, microRNA-409-3p. **(E)** Protein levels of ZEB1 in tumor tissues and adjacent normal tissues. OS, osteosarcoma. miR-NC, negative control microRNA mimics. Wt, wild type. Mut, mutant. ZEB1, Zinc-finger E-box-binding Homeobox-1. ^∗^*P* < 0.05, ^∗∗^*P* < 0.01 compared with the control group.

We then investigated the molecular mechanisms underlying the tumor suppression caused by miR-409-3p in OS by predicting its potential targets using bioinformatics analysis. The 3′ UTR of *ZEB1* was predicted to contain an miR-409-3p seed match at position 1280-1286 and has previously been reported as extensively upregulated in OS and participates in the regulation of OS tumorigenesis and progression (Shen et al., 2012; Li et al., 2016; Liu and Lin, 2016); therefore, we primarily focused on ZEB1 (Figure 3A) in this study. To validate the prediction, we performed luciferase reporter assays in HEK293T cells transfected with plasmids containing Mut and Wt ZEB 3′ UTR, along with miR-409-3p mimics or miR-NC. Luciferase activity was markedly downregulated in cells transfected with Wt ZEB1-3′ UTR and miR-409-3p mimics (Figure 3B, *P* < 0.01), however, no significant difference was observed in cells transfected with mutated ZEB1-3′ UTR and miR-409-3p mimics, suggesting that miR-409-3p could directly target the 3′ UTR of *ZEB1*. Additionally, RT-qPCR data showed that restoration of miR-409-3p expression led to down-regulation of *ZEB1* mRNA expression in MG63 and HOS cells (Figure 3C, *P* < 0.01). Moreover, Western blot analysis demonstrated that miR-409-3p reduced ZEB1 protein expression in MG63 and HOS cells (Figure 3D, *P* < 0.05). *In vivo* assay showed the protein levels in tumor tissues were significantly lower than those in adjacent normal tissues (Figure 3E, *P* < 0.01). To summarize, Our data demonstratedthat ZEB1 is potentially a direct target gene of miR-409-3p in OS.

### Upregulation of ZEB1 in OS Tissues and Negative Correlation of Its Expression With That of miR-409-3p

**FIGURE 4:**
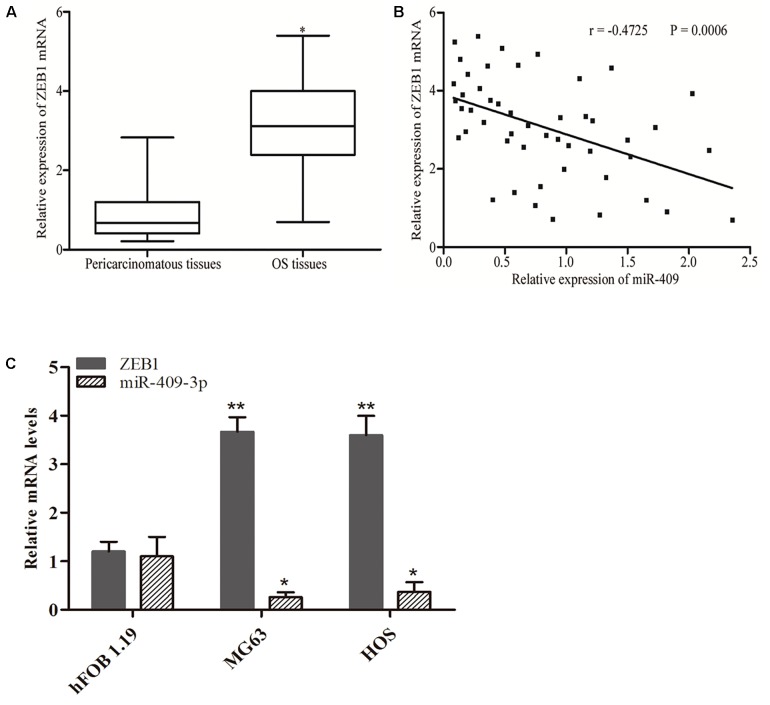
Inverse correlation between miR-409-3p and *ZEB1* mRNA expression levels in OS tissues. **(A)** RT-qPCR analysis showing that *ZEB1* mRNA levels were increased in OS tissues. **(B)** Spearman’s correlation analysis of the association between miR-409-3p and *ZEB1* mRNA in OS tissues. **(C)** Expressions of miR-409-3p and *ZEB1* mRNA in cell lines. miR-409, microRNA-409-3p. OS, osteosarcoma. ZEB1, Zinc-finger E-box-binding Homeobox-1. mRNA, message RNA. ^∗^*P* < 0.05, ^∗∗^*P* < 0.01 compared with the control group.

*ZEB1* is recognized as a direct target gene of miR-409-3p in OS; therefore, we next investigated whether miR-409-3p expression levels were negatively correlated with those of *ZEB1* in OS. Therefore, we performed RT-qPCR to evaluate *ZEB1* mRNA expression levels and found that they were higher in OS specimens than adjacent non-tumor tissues (Figure 4A, *P* < 0.05). Moreover, Spearman’s correlation analysis indicated an inverse relationship between miR-409-3p and *ZEB1* mRNA expression (Figure 4B, *r* = -0.4725, *P* = 0.0006) in OS tissue samples. As shown in Figure 4C, we observed higher expression of *ZEB1* mRNA and lower expression of miR-409-3p in tumor cell lines, when compared to normal cell line. Our results further confirm ZEB1 as a potential target of miR-409-3p in OS.

### Inhibition of ZEB1 Has Similar Effects to Those of miR-409-3p Overexpression in OS Cells

**FIGURE 5:**
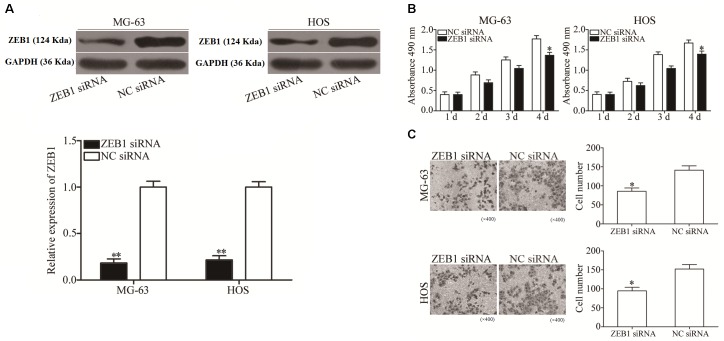
ZEB1 under-expression has similar effects to miR-409-3p over-expression on OS cell proliferation and invasion. **(A)** ZEB1 protein expression was detected in MG63 and HOS cells transfected with ZEB1 siRNA or NC siRNA. MTT **(B)** and cell invasion **(C)** assays were conducted in MG63 and HOS cells transfected with ZEB1 siRNA or NC siRNA. miR-409, microRNA-409-3p (magnification, × 200). OS, osteosarcoma. ZEB1, Zinc-finger E-box-binding Homeobox-1. mRNA, message RNA. siRNA, small interfering RNA. NC, negative control. ^∗^*P* < 0.05, ^∗∗^*P* < 0.01 compared with the control group.

To explore the biological roles of ZEB1 in response to miR-409-3p inhibition in OS, we investigated whether ZEB1 knockdown mimicked the effects of miR-409-3p overexpression in OS cells. ZEB1-targeting siRNA was used to knockdown ZEB1 expression in HOS and MG63 cells. As shown in Figure 5A, ZEB1 protein was successfully knocked down in HOS and MG63 cells transfected with ZEB1 siRNA (*P* < 0.01). MTT and cell invasion assays showed that knockdown of ZEB1 by the introduction of ZEB1 siRNA suppressed MG63 and HOS cell proliferation (Figure 5B, *P* < 0.05) and invasion (Figure 5C, *P* < 0.05), suggesting that negative regulation of ZEB1 may mediate the tumor suppressive effects of miR-409-3p in OS cells.

## Discussion

Dysregulation of miRNAs is a frequent event in various types of human cancer and has a pivotal role in the instigation of tumorigenesis and tumor progression where miRNAs can function as oncogenes or tumor suppressor genes (Fenger et al., 2014; Vanas et al., 2016). Furthermore, targeting miRNA with various types of chemically modified oligonucleotides has the potential to alter miRNA functions, providing a theoretical foundation for miRNA-based targeted therapy for specific human cancers (Trang et al., 2010; Imani et al., 2017; Tsai et al., 2017). Thus, research into the expression levels, biological roles, and fundamental molecular mechanisms of miRNAs has the potential to stimulate the development of novel approaches to the treatment of different types of cancer. Our data demonstrated that miR-409-3p expression levels were significantly down-regulated in OS tissues and cells relative to adjacent non-tumor tissues and a non-cancer osteoblastic cell line, respectively. Our observations are consistent with the findings of Ma et al. in breast cancer tissues and cell lines (Zheng et al., 2012). Additionally, reduced miR-409-3p expression levels were associated with clinical stage and distant metastasis in patients with OS, and our results also demonstrate that expression of miR-409-3p suppressed proliferation and invasion of OS cells. Furthermore, our data suggest that ZEB1 is a functional target of miR-409-3p in OS.

Recently, several studies have reported roles for abnormal miR-409-3p expression in the initiation and progression of various human cancers. For example, Josson et al. found that miR-409-3p expression was elevated in prostate cancer and that its re-expression in normal prostate fibroblasts resulted in a cancer-associated stroma-like phenotype, and miR-409-3p was released in extracellular vesicles to induce cancer initiation and epithelial-to-mesenchymal transition both *in vitro* and *in vivo* (Josson et al., 2015). Zheng et al. (2012) showed that miR-409-3p expression levels were decreased in gastric cancer and that they were negatively associated with tumor-node-metastasis stage and lymph node metastasis in patients with gastric cancer. Upregulation of miR-409-3p attenuated gastric cancer cell motility *in vitro* and decreased their ability to induce distal pulmonary metastases and peritoneal diffusion *in vivo* (Zheng et al., 2012). Tan et al. (2016) found that miR-409-3p was expressed at low levels in colon tumors and that its expression was negatively correlated with resistance to oxaliplatin. Ectopic expression of miR-409-3p improved the chemosensitivity of oxaliplatin-sensitive and oxaliplatin-resistant colon cancer cells (Tan et al., 2016). Therefore, miR-409-3p is a strong candidate for a new therapeutic target for the treatment of cancer because of its essential roles in cancer initiation and progression.

MiR-409-3p target identification is essential for understanding its potential functions in OS and developing novel targeted therapies for improving OS treatment. Potential miR-409-3p target genes have been previously reported; for example, Beclin-1 in colon cancer (Tan et al., 2016), radixin in gastric cancer (Zheng et al., 2012), and Ras suppressor 1 and stromal antigen 2 in prostate cancer (Josson et al., 2015). In the current study, we identified ZEB1 as a novel direct target of miR-409-3p in OS. Based on bioinformatics analysis, we predicted that *ZEB1* contains a miR-409-3p seed match at position 1280–1286 of the *ZEB1* 3′ UTR. Luciferase reporter assays demonstrated that miR-409-3p directly targeted the 3′ UTR of *ZEB1*. Furthermore, Western blot and RT-qPCR analysis indicated that endogenous miR-409-3p has a negative regulatory effect on *ZEB1* mRNA and protein expression in OS cells. Moreover, ZEB1 expression was high in OS tissues and inversely associated with that of miR-409-3p expression and knockdown of ZEB1 led to decreased OS cell proliferation and invasion, similar to miR-409-3p overexpression.

ZEB1, a member of the deltaEF1 family of two-handed zinc-finger transcription factors, maps to the short arm of human chromosome 10 (Zhang et al., 2019). ZEB1 expression is abnormally upregulated in various types of human cancer, including thyroid (Zhang et al., 2016), cervical (Ma et al., 2015), gastric (Jia et al., 2012), endometrial (Feng et al., 2014), and prostate (Drake et al., 2009) cancers. Accumulating evidence shows that ZEB1 has crucial roles during cancer initiation and progression (Kenney et al., 2011; Jia et al., 2012; Liu et al., 2012). In OS, Shen et al. reported that ZEB1 is highly expressed in tumor tissues and that its levels are significantly associated with lung metastasis. The signal network of ZEB1 involved in malignant transformation in various types of tumor is complicated. All of the upstream and downstream molecules participate in activating the signaling pathways in cell survival, senescence, chemosensitivity and immune escape, which may trigger the regulation of miR-409-3p. These findings suggest that inhibition of OS has the potential to be a novel and effective therapeutic target with the aim of curing this type of cancer. The limitations of this study include that we did not investigate the effects of ectopic ZEB1 over-expression on cell proliferation and invasion activity of miR-409-3p-expressing osteosarcoma cells and that the number of samples in this study is small thus multi-center trial is still needed.

## Conclusion

In conclusion, here we establish for the first time that miR-409-3p expression is down-regulated in OS tissues and cell lines. Decreased miR-409-3p expression levels were associated with clinical stage and distant metastasis. MiR-409-3p targets ZEB1, which may be associated with OS carcinogenesis and progression, leading to inhibition of OS cell proliferation and invasion. Thus, the miR-409-3p/ZEB1 axis can be considered a novel therapeutic target for OS treatment. Further research is needed to explore whether the potential of miR-409-3p/ZEB1 can be realized to treat OS.

## Author Contributions

These studies were conceived of and designed by all authors. Experiments were performed by LW and YZ. Data analysis, data interpretation, manuscript preparations were done by ZH, HG, KZ, XY, and JX.

## Conflict of Interest Statement

The authors declare that the research was conducted in the absence of any commercial or financial relationships that could be construed as a potential conflict of interest.

## Footnotes

1 http://www.targetscan.org/index.html

2 http://www.microrna.org
